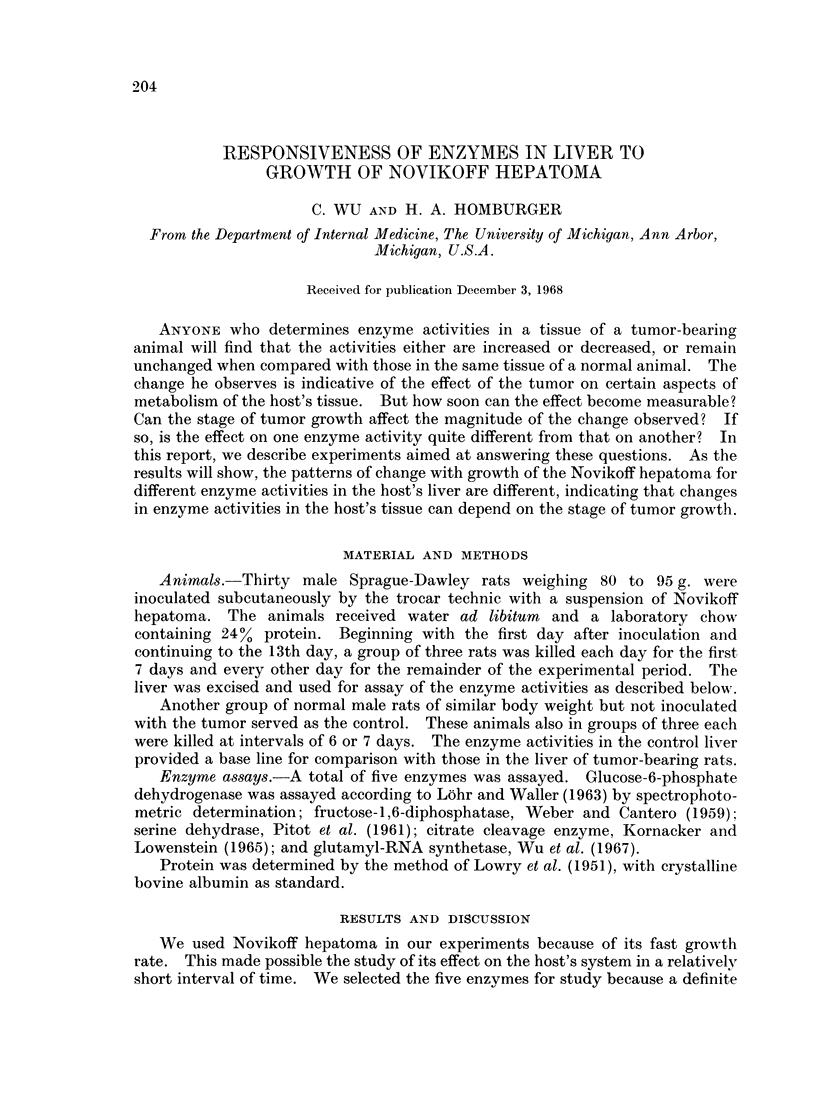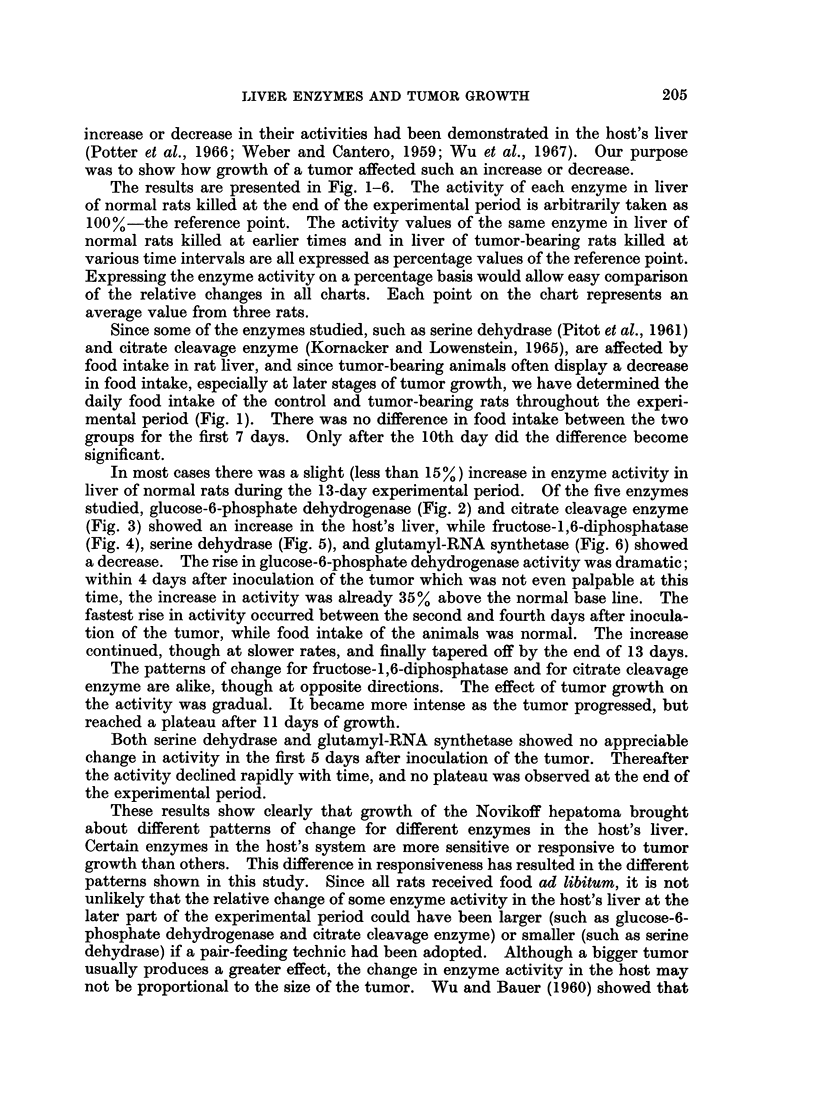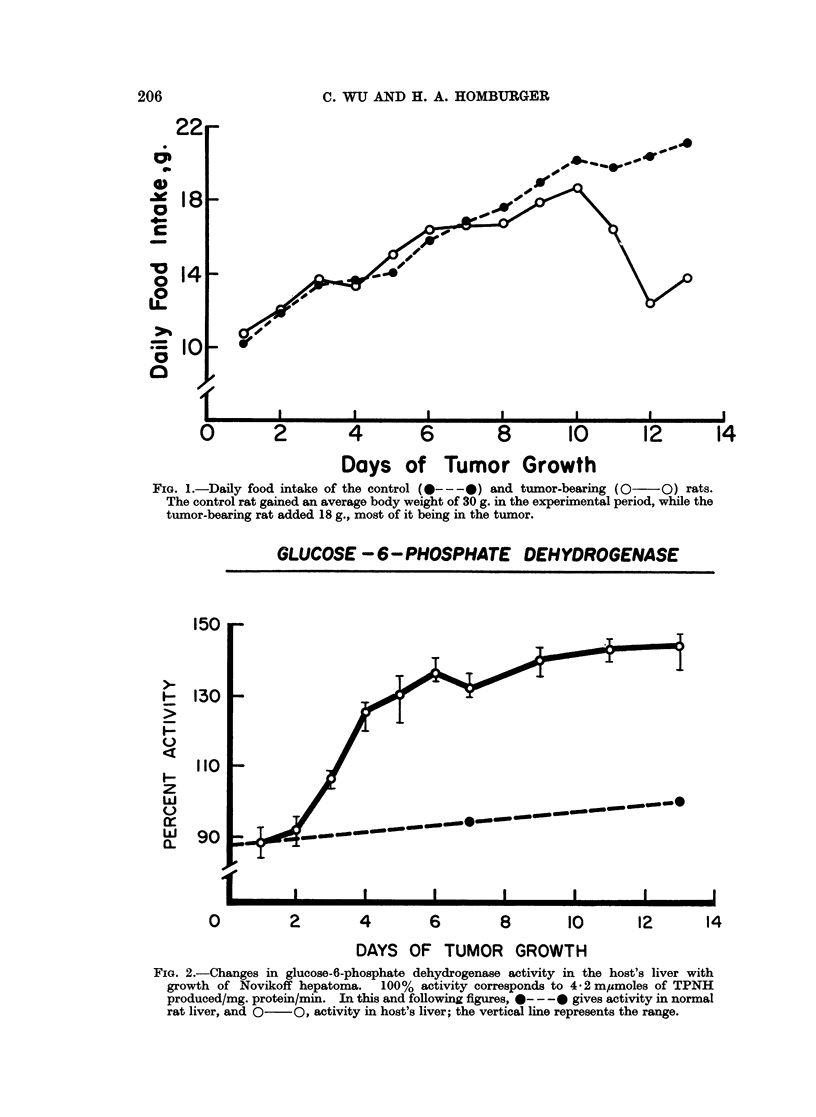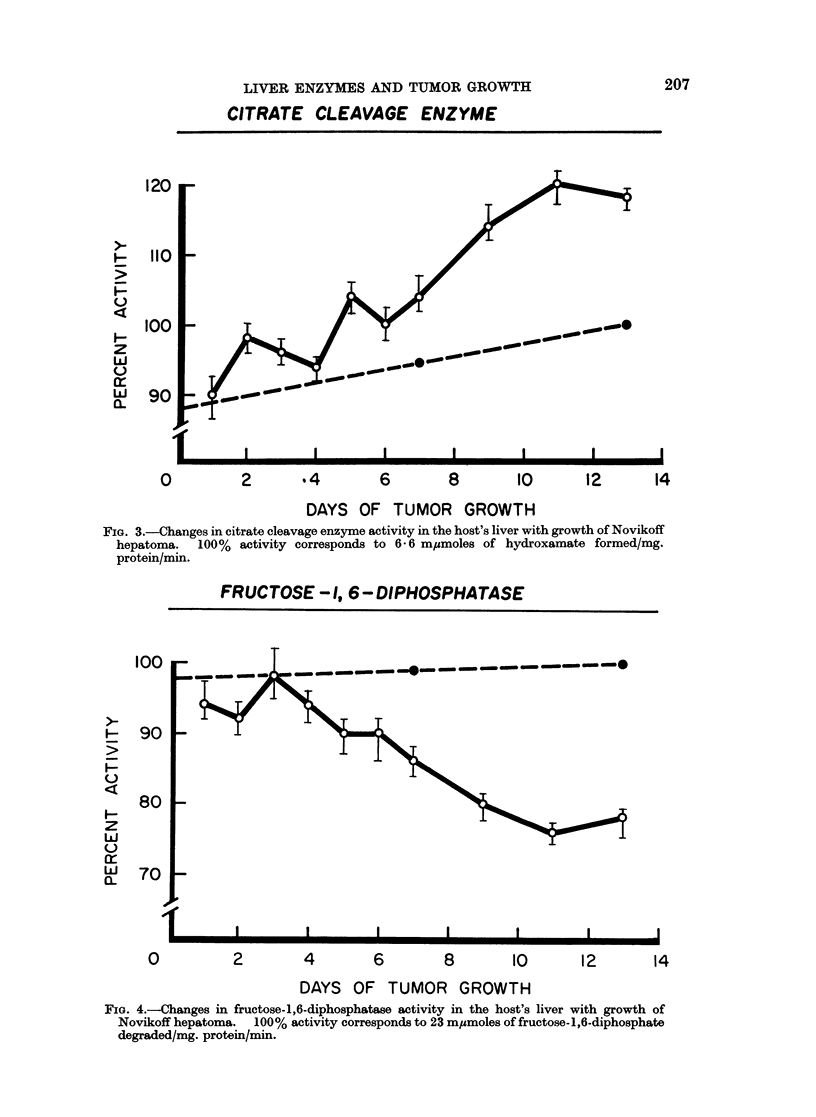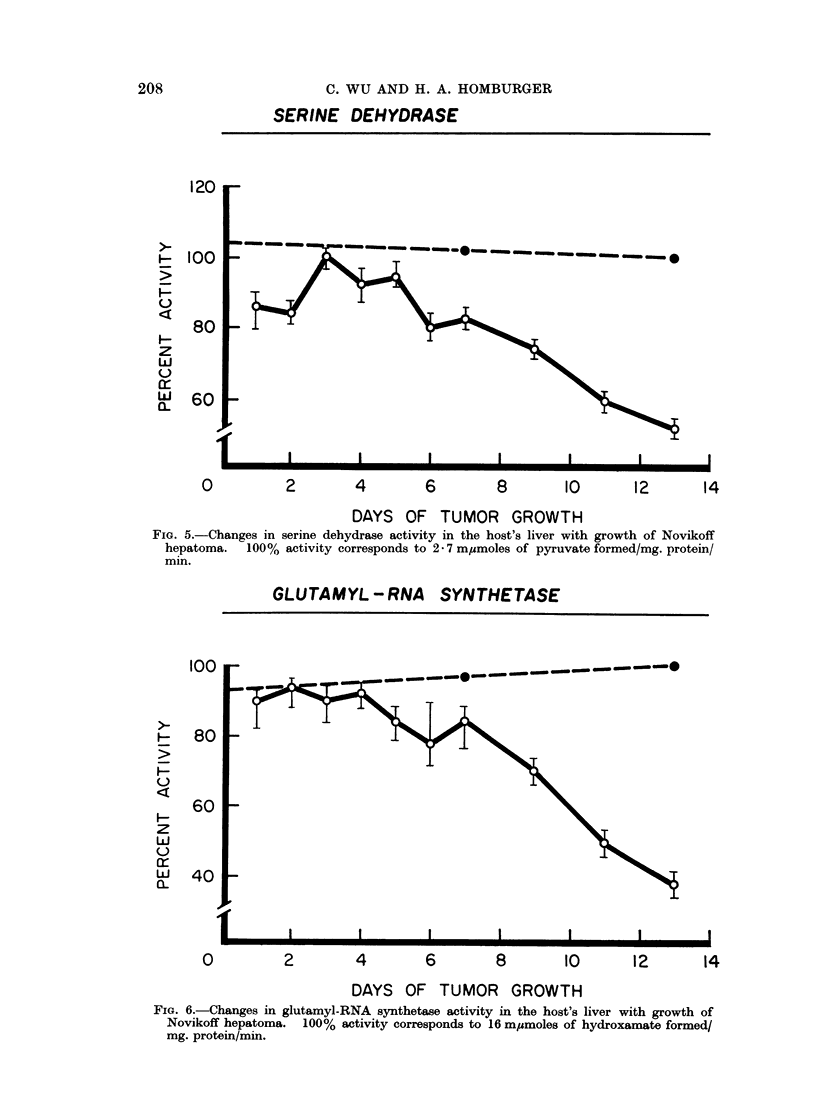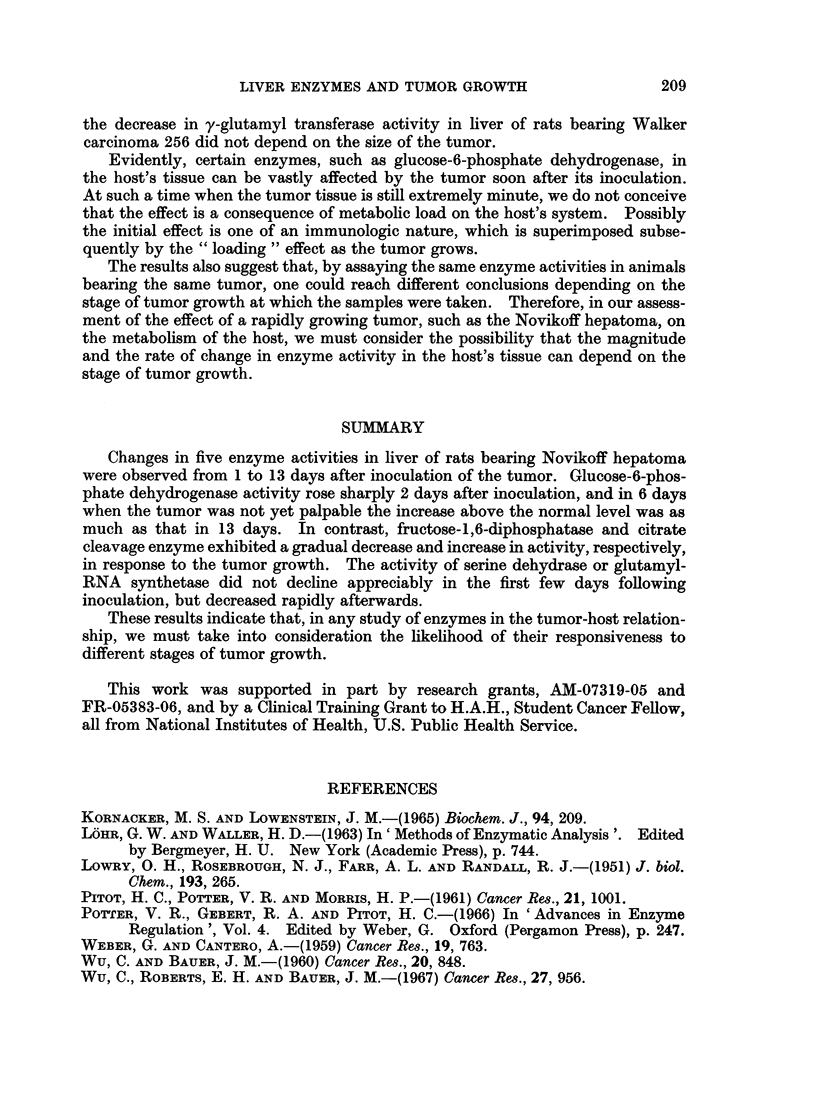# Responsiveness of enzymes in liver to growth of Novikoff hepatoma.

**DOI:** 10.1038/bjc.1969.29

**Published:** 1969-03

**Authors:** C. Wu, H. A. Homburger


### RESPONSIVENESS OF ENZYMES IN LIVER TO GROWTH OF NOVIKOFF HEPATOMA

C. WU AND H. A. HOMBURGER

*From the Department of Internal Medicine, The University of Michigan, Ann Arbor, Michigan, U.S.A.*

Received for publication December 3, 1968

ANYONE who determines enzyme activities in a tissue of a tumor-bearing animal will find that the activities either are increased or decreased, or remain unchanged when compared with those in the same tissue of a normal animal. The change he observes is indicative of the effect of the tumor on certain aspects of metabolism of the host's tissue. But how soon can the effect become measurable? Can the stage of tumor growth affect the magnitude of the change observed? If so, is the effect on one enzyme activity quite different from that on another? In this report, we describe experiments aimed at answering these questions. As the results will show, the patterns of change with growth of the Novikoff hepatoma for different enzyme activities in the host's liver are different, indicating that changes in enzyme activities in the host's tissue can depend on the stage of tumor growth.

### MATERIAL AND METHODS

*Animals.*—Thirty male Sprague-Dawley rats weighing 80 to 95 g. were inoculated subcutaneously by the trocar technic with a suspension of Novikoff hepatoma. The animals received water *ad libitum* and a laboratory chow containing 24% protein. Beginning with the first day after inoculation and continuing to the 13th day, a group of three rats was killed each day for the first 7 days and every other day for the remainder of the experimental period. The liver was excised and used for assay of the enzyme activities as described below.

Another group of normal male rats of similar body weight but not inoculated with the tumor served as the control. These animals also in groups of three each were killed at intervals of 6 or 7 days. The enzyme activities in the control liver provided a base line for comparison with those in the liver of tumor-bearing rats.

*Enzyme assays.*—A total of five enzymes was assayed. Glucose-6-phosphate dehydrogenase was assayed according to Löhr and Waller (1963) by spectrophotometric determination; fructose-1,6-diphosphatase, Weber and Cantero (1959); serine dehydrase, Pitot *et al.* (1961); citrate cleavage enzyme, Kornacker and Lowenstein (1965); and glutamyl-RNA synthetase, Wu *et al.* (1967).

Protein was determined by the method of Lowry *et al.* (1951), with crystalline bovine albumin as standard.

### RESULTS AND DISCUSSION

We used Novikoff hepatoma in our experiments because of its fast growth rate. This made possible the study of its effect on the host's system in a relatively short interval of time. We selected the five enzymes for study because a definite increase or decrease in their activities had been demonstrated in the host's liver (Potter *et al.*, 1966; Weber and Cantero, 1959; Wu *et al.*, 1967). Our purpose was to show how growth of a tumor affected such an increase or decrease.

The results are presented in Fig. 1–6. The activity of each enzyme in liver of normal rats killed at the end of the experimental period is arbitrarily taken as 100%—the reference point. The activity values of the same enzyme in liver of normal rats killed at earlier times and in liver of tumor-bearing rats killed at various time intervals are all expressed as percentage values of the reference point. Expressing the enzyme activity on a percentage basis would allow easy comparison of the relative changes in all charts. Each point on the chart represents an average value from three rats.

Since some of the enzymes studied, such as serine dehydrase (Pitot *et al.*, 1961) and citrate cleavage enzyme (Kornacker and Lowenstein, 1965), are affected by food intake in rat liver, and since tumor-bearing animals often display a decrease in food intake, especially at later stages of tumor growth, we have determined the daily food intake of the control and tumor-bearing rats throughout the experimental period (Fig. 1). There was no difference in food intake between the two groups for the first 7 days. Only after the 10th day did the difference become significant.

In most cases there was a slight (less than 15%) increase in enzyme activity in liver of normal rats during the 13-day experimental period. Of the five enzymes studied, glucose-6-phosphate dehydrogenase (Fig. 2) and citrate cleavage enzyme (Fig. 3) showed an increase in the host's liver, while fructose-1,6-diphosphatase (Fig. 4), serine dehydrase (Fig. 5), and glutamyl-RNA synthetase (Fig. 6) showed a decrease. The rise in glucose-6-phosphate dehydrogenase activity was dramatic; within 4 days after inoculation of the tumor which was not even palpable at this time, the increase in activity was already 35% above the normal base line. The fastest rise in activity occurred between the second and fourth days after inoculation of the tumor, while food intake of the animals was normal. The increase continued, though at slower rates, and finally tapered off by the end of 13 days.

The patterns of change for fructose-1,6-diphosphatase and for citrate cleavage enzyme are alike, though at opposite directions. The effect of tumor growth on the activity was gradual. It became more intense as the tumor progressed, but reached a plateau after 11 days of growth.

Both serine dehydrase and glutamyl-RNA synthetase showed no appreciable change in activity in the first 5 days after inoculation of the tumor. Thereafter the activity declined rapidly with time, and no plateau was observed at the end of the experimental period.

These results show clearly that growth of the Novikoff hepatoma brought about different patterns of change for different enzymes in the host's liver. Certain enzymes in the host's system are more sensitive or responsive to tumor growth than others. This difference in responsiveness has resulted in the different patterns shown in this study. Since all rats received food *ad libitum*, it is not unlikely that the relative change of some enzyme activity in the host's liver at the later part of the experimental period could have been larger (such as glucose-6-phosphate dehydrogenase and citrate cleavage enzyme) or smaller (such as serine dehydrase) if a pair-feeding technic had been adopted. Although a bigger tumor usually produces a greater effect, the change in enzyme activity in the host may not be proportional to the size of the tumor. Wu and Bauer (1960) showed that

FIG. 1.—Daily food intake of the control (●– – –●) and tumor-bearing (○———○) rats. The control rat gained an average body weight of 30 g. in the experimental period, while the tumor-bearing rat added 18 g., most of it being in the tumor.

FIG. 2.—Changes in glucose-6-phosphate dehydrogenase activity in the host's liver with growth of Novikoff hepatoma. 100% activity corresponds to 4·2 mμmoles of TPNH produced/mg. protein/min. In this and following figures, ●– – –● gives activity in normal rat liver, and ○———○, activity in host's liver; the vertical line represents the range.

Fig. 3.—Changes in citrate cleavage enzyme activity in the host's liver with growth of Novikoff hepatoma. 100% activity corresponds to 6·6 mμmoles of hydroxamate formed/mg. protein/min.

Fig. 4.—Changes in fructose-1,6-diphosphatase activity in the host's liver with growth of Novikoff hepatoma. 100% activity corresponds to 23 mμmoles of fructose-1,6-diphosphate degraded/mg. protein/min.

FIG. 5.—Changes in serine dehydrase activity in the host's liver with growth of Novikoff hepatoma. 100% activity corresponds to 2·7 mμmoles of pyruvate formed/mg. protein/min.

FIG. 6.—Changes in glutamyl-RNA synthetase activity in the host's liver with growth of Novikoff hepatoma. 100% activity corresponds to 16 mμmoles of hydroxamate formed/mg. protein/min.

the decrease in γ-glutamyl transferase activity in liver of rats bearing Walker carcinoma 256 did not depend on the size of the tumor.

Evidently, certain enzymes, such as glucose-6-phosphate dehydrogenase, in the host's tissue can be vastly affected by the tumor soon after its inoculation. At such a time when the tumor tissue is still extremely minute, we do not conceive that the effect is a consequence of metabolic load on the host's system. Possibly the initial effect is one of an immunologic nature, which is superimposed subsequently by the " loading " effect as the tumor grows.

The results also suggest that, by assaying the same enzyme activities in animals bearing the same tumor, one could reach different conclusions depending on the stage of tumor growth at which the samples were taken. Therefore, in our assessment of the effect of a rapidly growing tumor, such as the Novikoff hepatoma, on the metabolism of the host, we must consider the possibility that the magnitude and the rate of change in enzyme activity in the host's tissue can depend on the stage of tumor growth.

### SUMMARY

Changes in five enzyme activities in liver of rats bearing Novikoff hepatoma were observed from 1 to 13 days after inoculation of the tumor. Glucose-6-phosphate dehydrogenase activity rose sharply 2 days after inoculation, and in 6 days when the tumor was not yet palpable the increase above the normal level was as much as that in 13 days. In contrast, fructose-1,6-diphosphatase and citrate cleavage enzyme exhibited a gradual decrease and increase in activity, respectively, in response to the tumor growth. The activity of serine dehydrase or glutamyl-RNA synthetase did not decline appreciably in the first few days following inoculation, but decreased rapidly afterwards.

These results indicate that, in any study of enzymes in the tumor-host relationship, we must take into consideration the likelihood of their responsiveness to different stages of tumor growth.

This work was supported in part by research grants, AM-07319-05 and FR-05383-06, and by a Clinical Training Grant to H.A.H., Student Cancer Fellow, all from National Institutes of Health, U.S. Public Health Service.

### REFERENCES

KORNACKER, M. S. AND LOWENSTEIN, J. M.—(1965) *Biochem. J.*, **94**, 209.
LÖHR, G. W. AND WALLER, H. D.—(1963) In ' Methods of Enzymatic Analysis '. Edited by Bergmeyer, H. U. New York (Academic Press), p. 744.
LOWRY, O. H., ROSEBROUGH, N. J., FARR, A. L. AND RANDALL, R. J.—(1951) *J. biol. Chem.*, **193**, 265.
PITOT, H. C., POTTER, V. R. AND MORRIS, H. P.—(1961) *Cancer Res.*, **21**, 1001.
POTTER, V. R., GEBERT, R. A. AND PITOT, H. C.—(1966) In ' Advances in Enzyme Regulation ', Vol. 4. Edited by Weber, G. Oxford (Pergamon Press), p. 247.
WEBER, G. AND CANTERO, A.—(1959) *Cancer Res.*, **19**, 763.
WU, C. AND BAUER, J. M.—(1960) *Cancer Res.*, **20**, 848.
WU, C., ROBERTS, E. H. AND BAUER, J. M.—(1967) *Cancer Res.*, **27**, 956.